# Supplementary material for: Gene Panel Testing for Breast Cancer Reveals Differential Effect of Prior BRCA1/2 Probability
Source: Cancers (Basel). 2021 Aug 18;13(16):4154. doi: 10.3390/cancers13164154 (PMC8394249; doi:10.3390/cancers13164154)
Supplement: Supplementary file 1 [file cancers-13-04154-s001.zip › cancers-1334905-supplementary.pdf]

Article

Gene Panel Testing for Breast Cancer Reveals Differential Effect of Prior *BRCA1*/2 Probability

D. Gareth Evans [1,2,3,4,†,*], Elke M. van Veen [1,2,†], Emma R. Woodward [1,2], Elaine F. Harkness [3], Jamie M. Ellingford [1,2], Naomi L. Bowers [1], Andrew J. Wallace [1], Sacha Howell [3,4,5], Anthony Howell [3,4], Fiona Lalloo [1], William G. Newman [1,2] and Miriam J. Smith [1,2]

[1] NW Genomic Laboratory hub, Manchester Centre for Genomic Medicine, Manchester University Hospitals NHS Foundation Trust, Manchester M13 9WL, UK; Elke.van-Veen@manchester.ac.uk (E.M.v.V.); Emma.Woodward@mft.nhs.uk (E.R.W.); jamie.ellingford@manchester.ac.uk (J.M.E.); Naomi.Bowers@mft.nhs.uk (N.L.B.); andrew.wallace@mft.nhs.uk (A.J.W.); Fiona.Lalloo@mft.nhs.uk (F.L.); William.newman@manchester.ac.uk (W.G.N.); miriam.smith@manchester.ac.uk (M.J.S.)

[2] Division of Evolution and Genomic Sciences, School of Biological Sciences, Faculty of Biology, Medicine and Health, University of Manchester, Manchester Academic Health Science Centre, Manchester, UK

[3] Prevent Breast Cancer Centre, Wythenshawe Hospital Manchester Universities Foundation Trust, Wythenshawe, Manchester M23 9LT, UK; Elaine.F.Harkness@manchester.ac.uk (E.F.H.); sacha.howell@manchester.ac.uk (S.H.); anthony.howell@manchester.ac.uk (A.H.)

[4] Manchester Breast Centre, The Christie NHS Foundation Trust, Wilmslow Road, Manchester M20 4BX, UK

[5] Division of Cancer Sciences, Faculty of Biology, Medicine and Health, University of Manchester, Manchester Academic Health Science Centre, Manchester, UK

* Correspondence: Gareth.Evans@mft.nhs.uk; Tel.:

Supplementary Tables

Table S1. Results from *MSH6* testing of 871 women with breast cancer (524 from PROCAS) and 1443 controls.

PROCAS ID	Breast cancer	Gene	Variant annotation	Tumour type after entry	Grade	ER	PR	HER2	MSS
PRC/GMR-14418	Yes	MSH6	c.718C>T; p.(Arg240*)	IDC with DCIS	3	Negative	Negative	Negative	15
PRC/GMR-32914	Yes	MSH6	c.2910G>A; p.(Trp970*)	Invasive Lobular Carcinoma	2	Negative	Negative	Positive	1
PRC/GMR-7293	No	MSH6	c.2550C>G; p.(Tyr850*)	-	-	-	-	-	-

Table S2. Panel testing results for women with a personal (n=25) or family (n=109) history of ovarian cancer.

Genes	2--8	9--10	11--12	13--14	15--19	20--24	25-29	30-39	40+	total
panel positive	2	0	0	0	1	1	2	2	0	8
total	9	3	3	8	18	22	29	28	14	134
%	22.22%	0.00%	0.00%	0.00%	5.56%	4.55%	6.90%	7.14%	0.00%	5.97%
ATM	0	0	0	0	0	0	0	1	0	1
CHEK2	0	0	0	0	1	1	0	1	0	3
TP53	0	0	0	0	0	0	0	0	0	0
PALB2	1	0	0	0	0	0	1	0	0	2
NBN	0	0	0	0	0	0	0	0	0	0
RAD51C	0	0	0	0	0	0	1	0	0	1
RAD51D	0	0	0	0	0	0	0	0	0	0
BARD1	1	0	0	0	0	0	0	0	0	1
All BRCA tested										
BRCA1	3	0	2	1	7	26	31	52	49	171
BRCA2	1	1	3	1	17	28	28	28	17	124
Total tested	53	25	48	61	173	179	160	127	79	905
% *BRCA1/2*	7.55%	4.00%	10.42%	3.28%	13.87%	30.17%	36.88%	62.99%	83.54%	32.60%
Panel with no prescreen	3	3	2	3	5	8	7	10	5	46.00
BRCA1	0	0	0	0	0	1	1	4	4	10.00
BRCA2	0	0	0	0	2	0	2	2	1	7.00
% *BRCA1/2*	0.00%	0.00%	0.00%	0.00%	40.00%	12.50%	42.86%	60.00%	100.00%	36.96%

Table S3. Panel testing results for women with a personal (n=25) history of breast and ovarian cancer.

Genes	2--8	9--10	11--12	13--14	15--19	20--24	25-29	30-39	40+	total
panel positive	0	0	0	0	0	0	1	1	0	2
total	0	0	0	0	6	3	7	7	2	25
%	0.00%	0.00%	0.00%	0.00%	0.00%	0.00%	14.29%	14.29%	0.00%	8.00%
ATM	0	0	0	0	0	0	0	1	0	1
CHEK2	0	0	0	0	0	0	0	0	0	0
TP53	0	0	0	0	0	0	0	0	0	0
PALB2	0	0	0	0	0	0	0	0	0	0
NBN	0	0	0	0	0	0	0	0	0	0
RAD51C	0	0	0	0	0	0	1	0	0	1
RAD51D	0	0	0	0	0	0	0	0	0	0
BARD1	0	0	0	0	0	0	0	0	0	0
All BRCA tested										
BRCA1	0	0	1	0	4	11	8	16	19	59
BRCA2	0	1	3	0	8	12	4	6	4	38
Total tested	5	4	23	19	56	52	31	32	24	246
% *BRCA1/2*	0.00%	25.00%	17.39%	0.00%	21.43%	44.23%	38.71%	68.75%	95.83%	39.43%
Panel with no prescreen	0	0	0	0	1	0	2	0	0	3
BRCA1	0	0	0	0	0	0	1	0	0	1
BRCA2	0	0	0	0	0	0	0	0	0	0
% *BRCA1/2*					0.00%		50.00%			33.33%
